# Supplementary figures and images for: Deletion of the Plasmodium falciparum exported protein PTP7 leads to Maurer’s clefts vesiculation, host cell remodeling defects, and loss of surface presentation of EMP1
Source: PLoS Pathog. 2022 Aug 5;18(8):e1009882. doi: 10.1371/journal.ppat.1009882 (PMC9385048; doi:10.1371/journal.ppat.1009882)

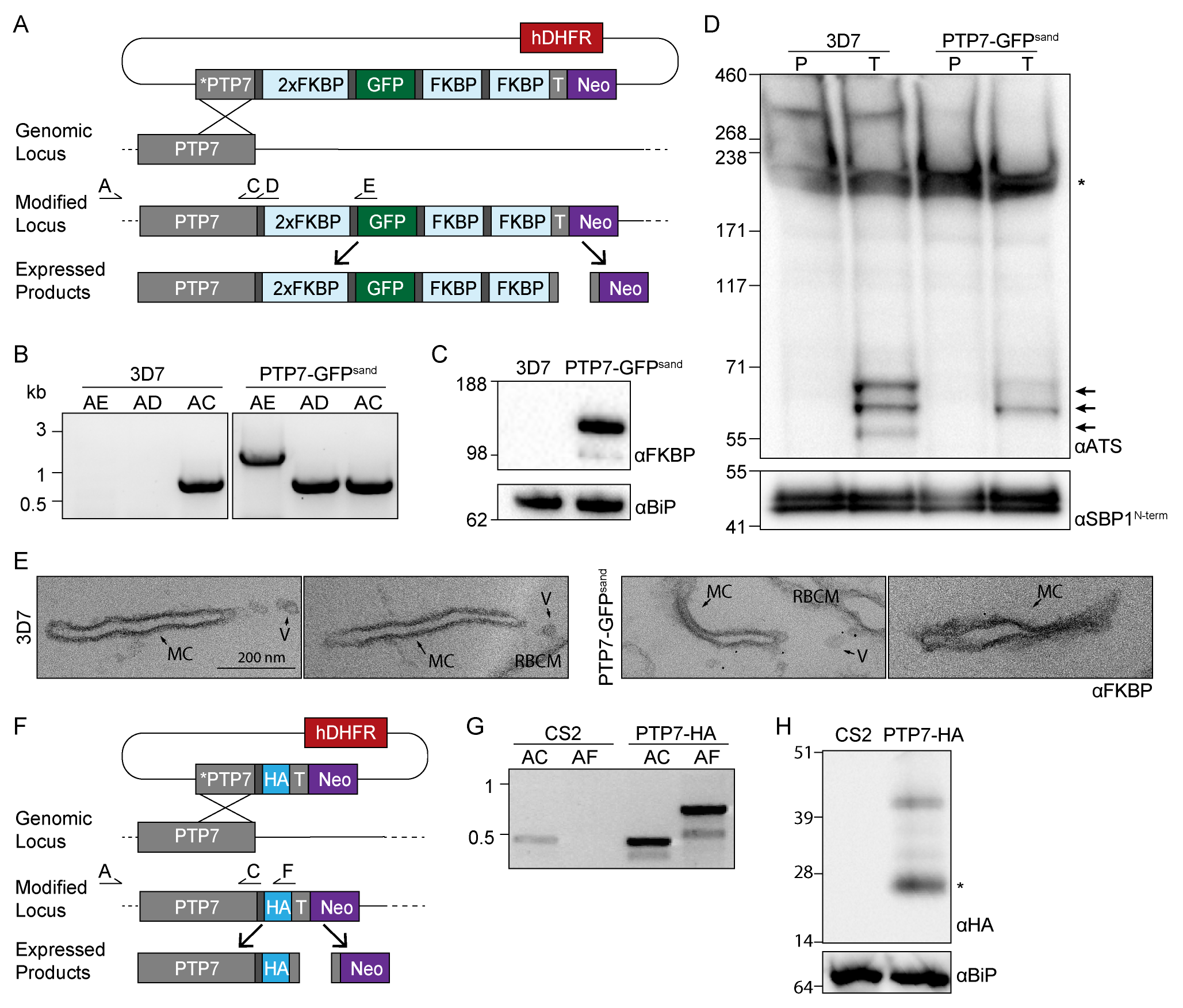

Supplement: S1 Fig — (A) Schematic illustrating selection linked integration of 2xFKBP-GFP-2xFKBP tag (GFPsand) at the 3’ end of the PTP7 locus. Dark gray rectangles: linkers; T: T2A skip peptide; Neo: neomycin selectable marker; hDHFR selectable marker; *PTP7: PTP7 homology region with 5’ stop codon; crossed lines: homologous cross over event; letters (A-E) and half arrows: primer locations. (B) Confirmation of correct integration of the PTP7-GFPsand plasmid into the endogenous locus. (C) Immunoblot of parent line (3D7) and PTP7-GFPsand cell lysates probed with αFKBP. Loading control, αBiP, expected size of ~62 kDa. (D) Immunoblots of the TritonX-100 insoluble, SDS soluble fraction of surface trypsinized infected RBCs shown. 3D7: parent line. High molecular weight band is full length EMP1 recognized by αATS. Bands annotated with an asterisk indicate spectrin cross-reactivity expected at approximately 225 kDa for polypeptides [75] and 65 kDa for spectrin degradation products [76]. Arrows indicate cleaved EMP1 species where intracellular ATS regions were protected during trypsin incubation. Loading control, αSBP1, is also an experimental control, as breaching of the infected red blood cell membrane during trypsinization would cleave the Maurer’s cleft protein SBP1. SBP1 expected molecular weight of ~50 kDa [28]. (E) Representative immuno-TEM micrographs of 20–32 hpi infected RBCs permeabilized with Equinatoxin-II. Cells were probed for FKBP followed by immunogold secondary labeling. (F) Schematic illustrating selection linked integration of HA tag at the 3’ end of the PTP7 locus. (G) Confirmation of correct integration of the PTP7-GFPsand plasmid into the endogenous locus. (H) Immunoblot of PTP7-HA and parent line CS2 cell lysates probed with αHA, fusion protein expected to run anomalously high due to low complexity regions (Fig 2C) ~41 kDa. Asterisk indicates likely degradation product. Loading control αBiP, expected size of ~62 kDa. (TIF) [file ppat.1009882.s001.tif]

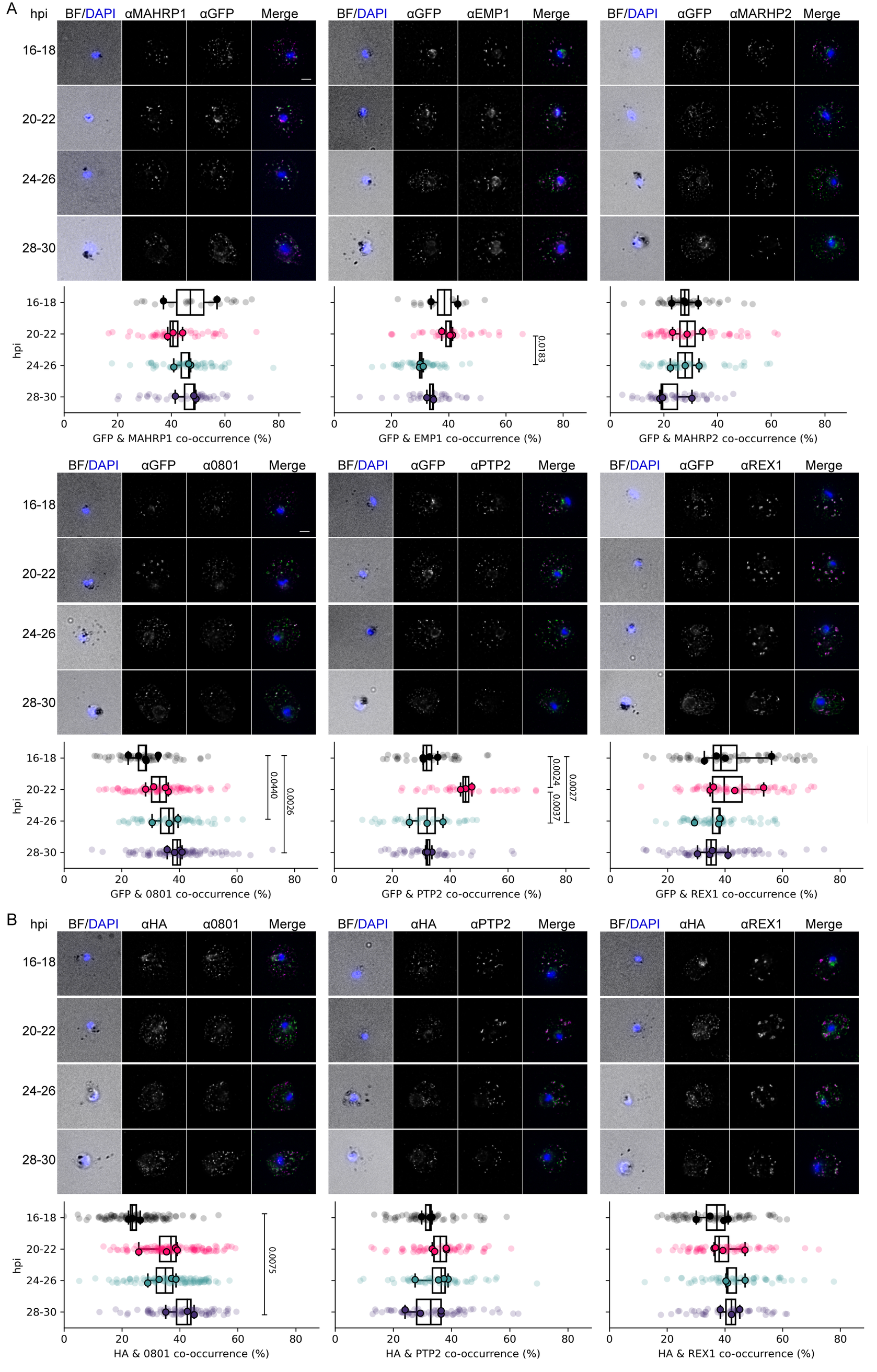

Supplement: S2 Fig — Indirect immunofluorescence assays of paraformaldehyde fixed PTP7-GFPsand (A) and PTP7-HA (B) infected RBCs probed with the antibodies indicated. Cells were synchronized to a 2-hour window and measured every 4 hours from 16 to 28 hpi. Scale bar, 2 μm. For quantitation of the ratio of PTP7 positive puncta to total puncta per antibody set, images were maximum projected and the parasitophorous vacuole signal was excluded. Data displayed are mean ± SD of the means per biological repeat. P-values determined by Tukey HSD multiple comparison tests (n = 2–4), n values are biological repeats. (TIF) [file ppat.1009882.s002.tif]

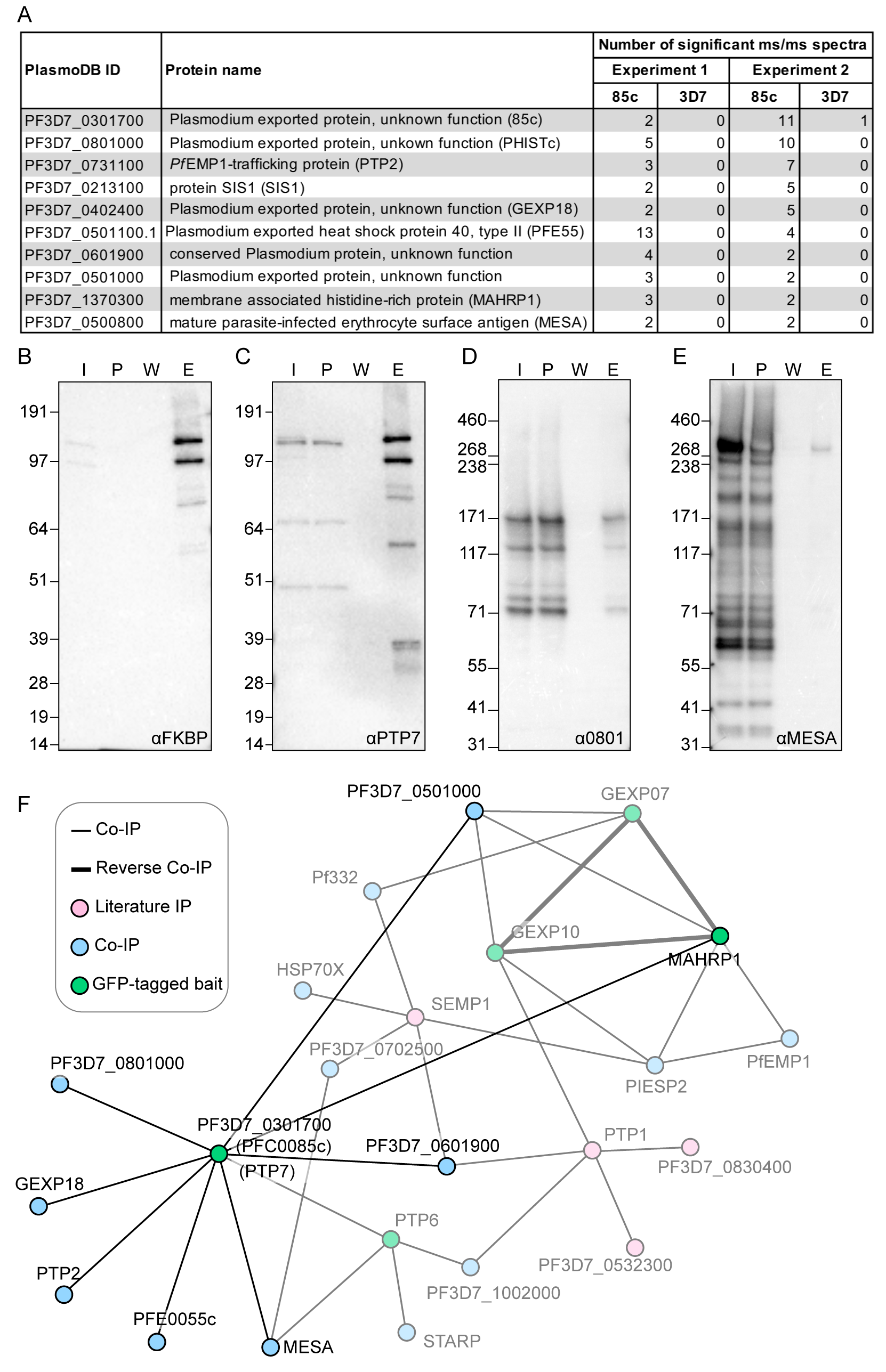

Supplement: S3 Fig — (A) Summary of proteins 3-fold enriched relative to parent-line controls identified by PTP7-GFPsand immunoprecipitation using GFP-Trap (n = 2). (B) Immunoprecipitation of PTP7-GFPsand parasite lysate using GFP-Trap including I, input; P, post-GFP-Trap; W, resin wash; E, eluate. Expected size for PTP7 tagged with 4xFKBP 1xGFP domains is 108 kDa. Blots probed with primary antibodies against FKBP (B), PTP7 (C), 0801 (D) and MESA (E) and detected with HRP conjugated secondary antibodies. (F) Network map of exported P. falciparum co-immunoprecipitants identified from PTP7-GFP bait, overlayed with network established in [25,32,38]. (TIF) [file ppat.1009882.s003.tif]

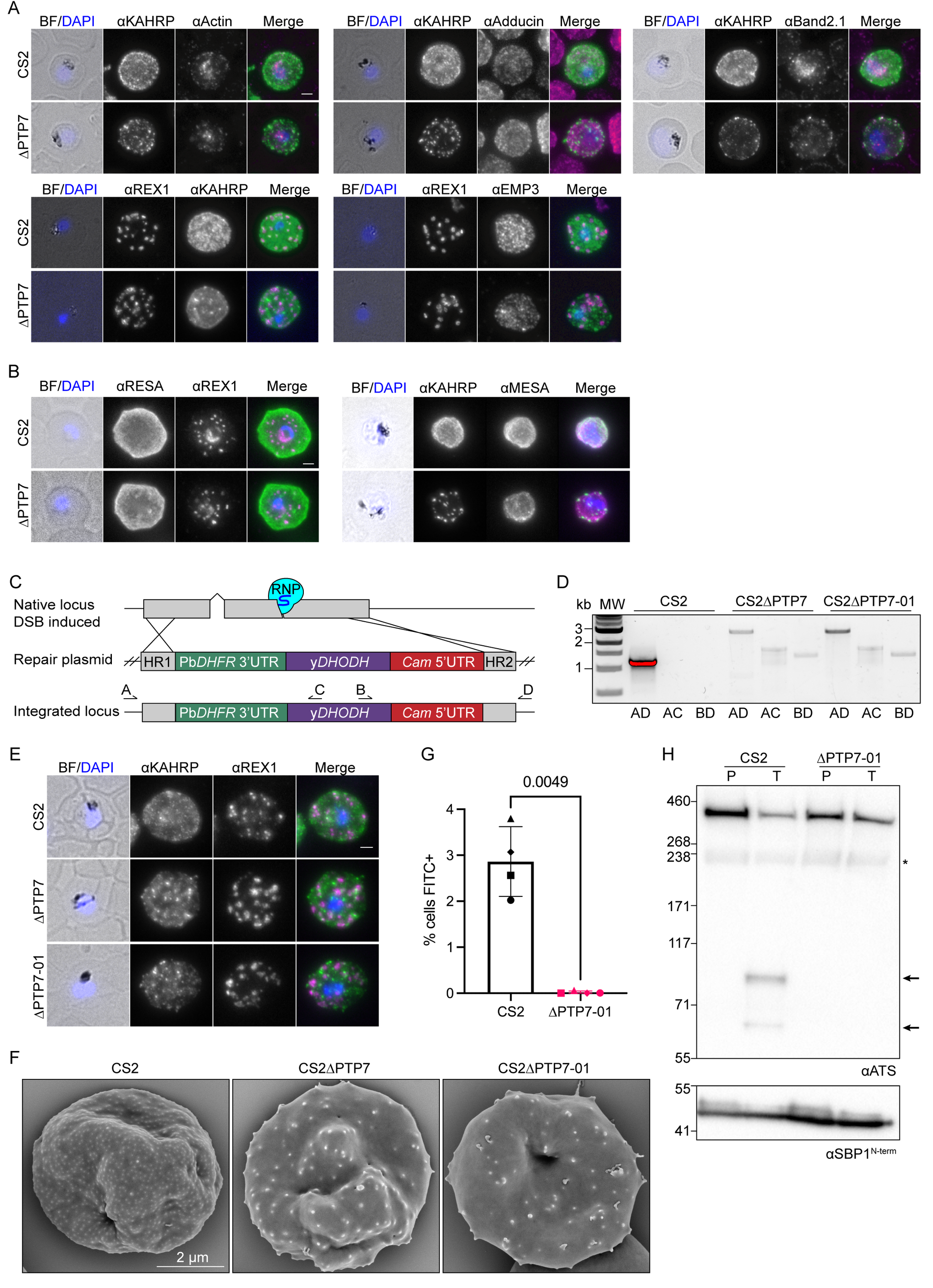

Supplement: S4 Fig — (A-B) Indirect immunofluorescence assays of cells fixed at an acetone methanol ratio of 1:1 (A) or 9:1 (B) then probed with antibodies as indicated above each panel. Bright field (BF) and DAPI stained DNA (blue) images are merged. Green and magenta are used for the merged images of the two antibodies. Scale bar, 2 μm. (C) Schematic outlining the gene disruption strategy as illustrated in Fig 2. (D) PCR products of CS2 and independent knockout CS2ΔPTP7_01 genomic DNA confirming disruption of the ptp7 locus. Red indicates saturated pixels. (E) Indirect immunofluorescence assays of cells fixed at an acetone methanol ratio 9:1 then probed with antibodies as indicated above each panel. Bright field (BF) and DAPI stained DNA (blue) images are merged. Green and magenta are used for the merged images of the two antibodies. Scale bar, 2 μm. (F) Mid-trophozoite stage infected RBCs were fixed in 2.5% glutaraldehyde/PBS and prepared for SEM of the exterior surface. (G) Flow cytometry analysis of infected RBCs labeled with antibodies for the ectodomain of var2CSA followed by secondary antibodies and tertiary antibodies conjugated to Alexa Fluor 647. Samples were run in technical duplicates. Data displayed are mean fluorescence values ± SD for each biological repeat (n = 4 per cell line). (H) Trypsin cleavage assay of truncation lines. Membranes were probed with αATS and the loading/experimental control αSBP1. P: PBS mock treatment; T: Trypsin treated samples; asterisk: spectrin cross-reactivity band; arrows: EMP1 cleavage products. Loading control and experimental control, SBP1, expected molecular weight is ~50 kDa. (TIF) [file ppat.1009882.s004.tif]

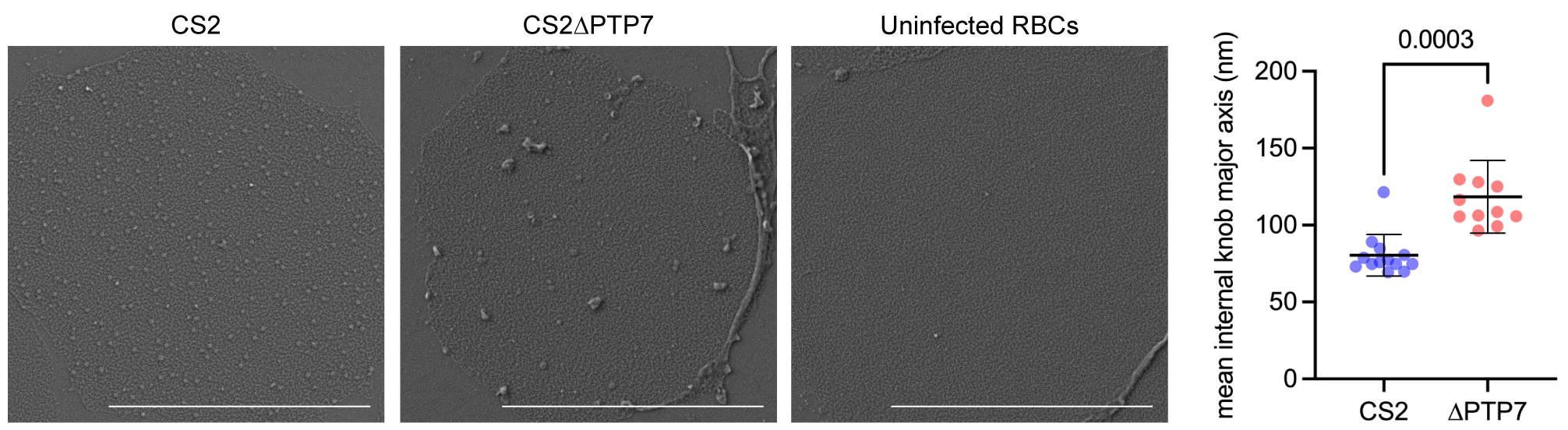

Supplement: S5 Fig — Early to mid-trophozoite cultures were adhered to lectin functionalized slides and sheared off with hypotonic buffer to reveal the cytoplasmic face of the infected red blood cell footprint which was then fixed, dehydrated, coated with gold, and imaged using scanning electron microscopy. Knobs are indicated by inverted discs. Data displayed are mean ± SD (CS2 n = 14; ΔPTP7 n = 12). P-values determined by Welch’s t-test, n values are individual cells from ≥2 biological repeats. (TIF) [file ppat.1009882.s005.tif]

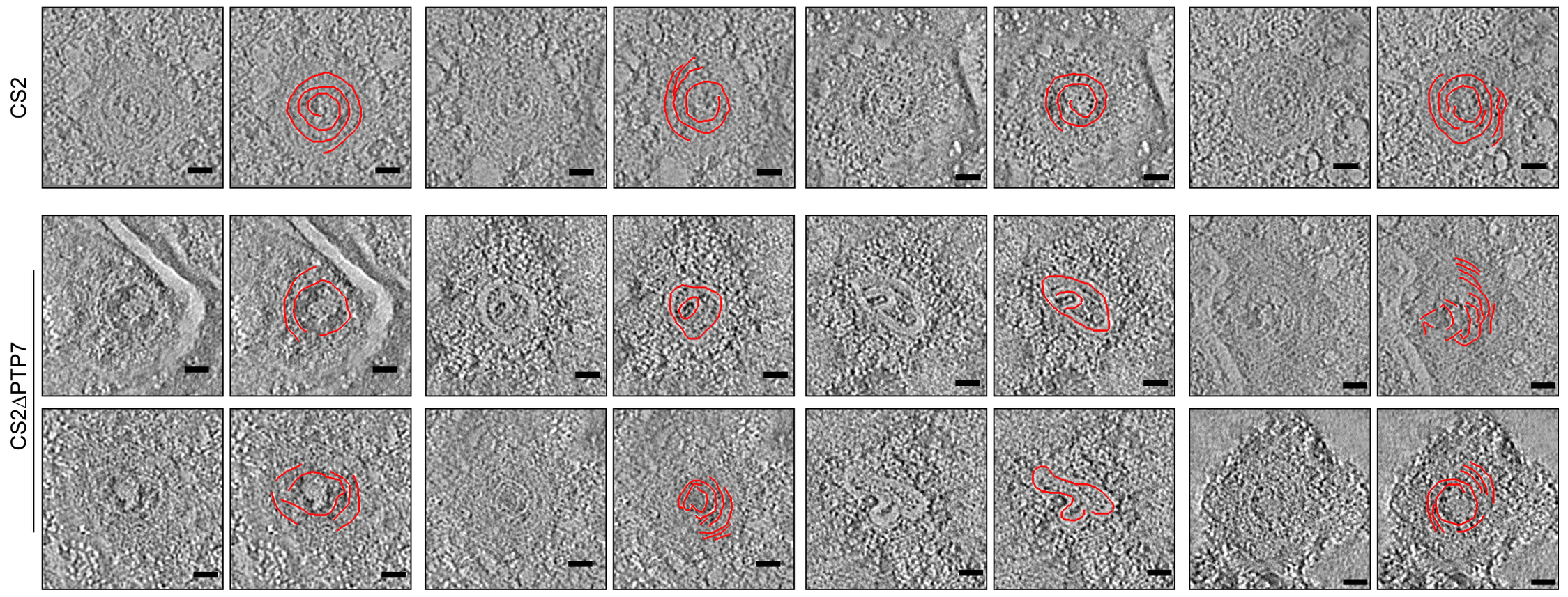

Supplement: S6 Fig — Polylysine functionalized grids were incubated with infected RBCs then lysed and imaged. Tomograms reveal the spiral structure underlying the infected red blood cell membrane associated with knobs [39]. Scale bar, 20 nm. (TIF) [file ppat.1009882.s006.tif]

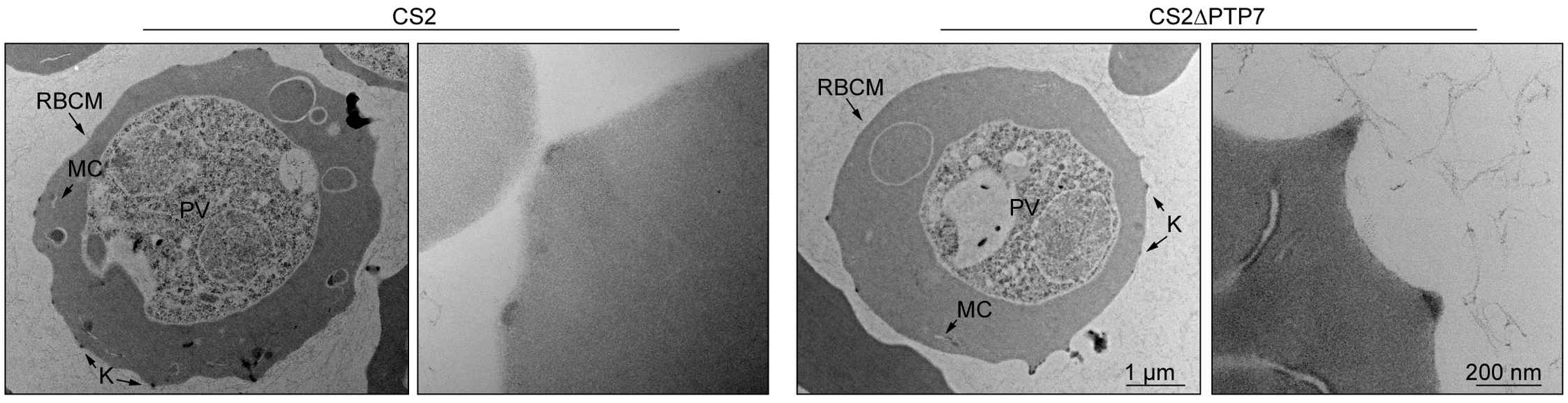

Supplement: S7 Fig — Mid-trophozoite stage infected red blood cells. (TIF) [file ppat.1009882.s007.tif]

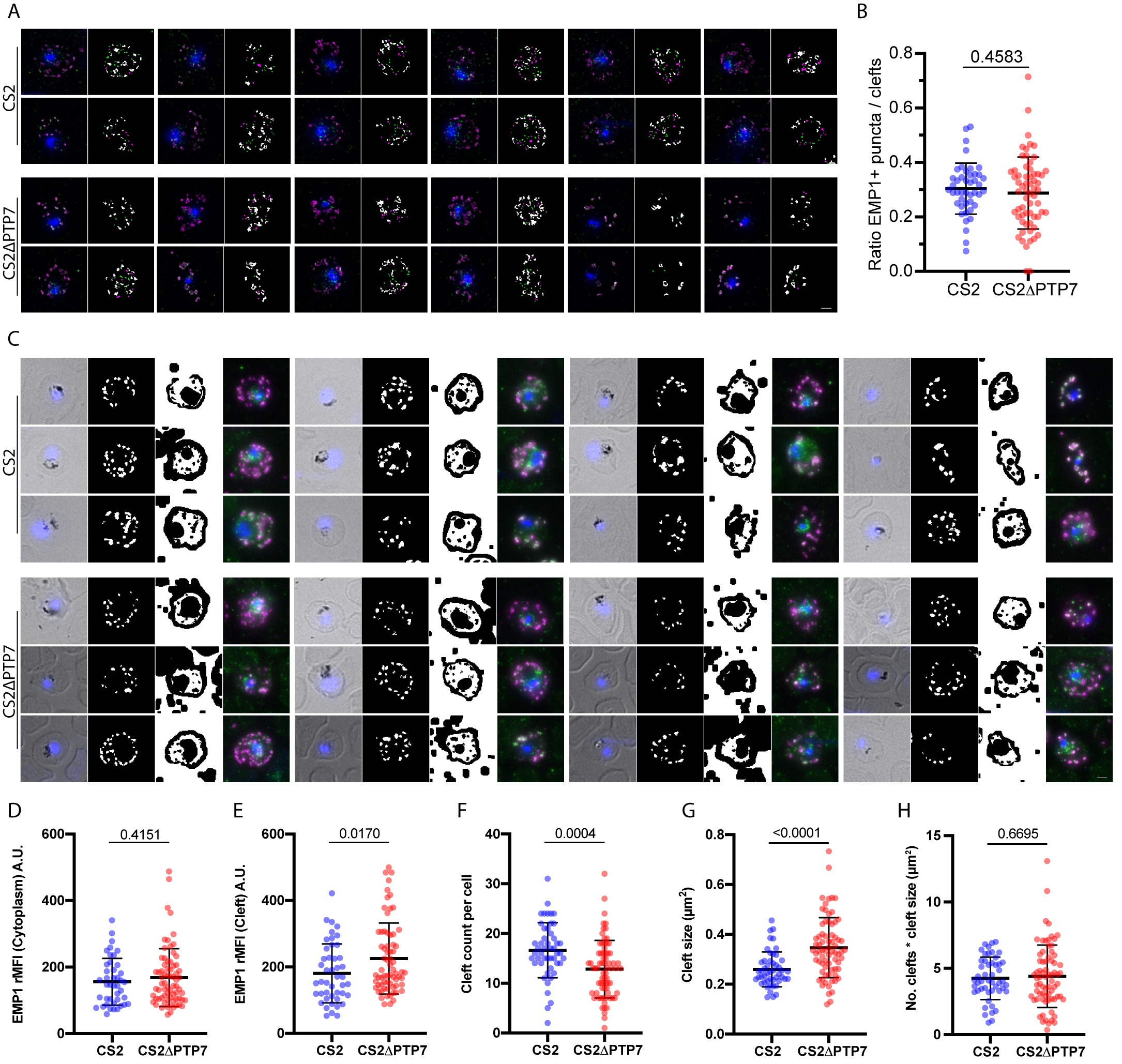

Supplement: S8 Fig — (A) IFA channels probed with αATS and αREX1 were Huygens deconvolved to resolve cytoplasmic αATS puncta and clefts indicated by αREX1 signal. A FIJI Macro script counts the αATS only, αREX1 only, and both particles to generate: a proof, including the merge image (color assignment as in (Fig 4A)) and results image with EMP1 only (green), REX1 only (magenta) and both (white) depicted. (B) Ratio of EMP1-only particles to clefts. Data displayed are mean ± SD (CS2 n = 44; CS2ΔPTP7 n = 63). (C-H) Quantitation of αATS signal distribution and cleft features. (C) Additional examples of the cleft and cytoplasm masks used to quantify the fluorescence intensities of these compartments. Bright field (BF) and DNA stain DAPI merged for reference. The ‘Cleft mask’ depicts where αATS signal was classified as ‘cleft’. The ‘Cytoplasm’ mask illustrates i) the internal object used to determine αATS cytoplasmic signal and ii) the external space where αATS signal was measured as a background mean gray value control. The merge illustrates the merged IFA channels (color assignment as in (Fig 4A)). Mean gray values in the cleft and cytoplasm compartments were background subtracted. Data analysis performed is indicated in the y-axis. Data displayed are mean ± SD ((D) CS2 n = 42; CS2ΔPTP7 n = 71. (E) CS2 n = 45; CS2ΔPTP7 n = 69. (F) CS2 n = 41; CS2ΔPTP7 n = 68. (G) CS2 n = 49; CS2ΔPTP7 n = 72. (H) CS2 n = 49; CS2Δ85c n = 72.). P-values determined by Welch’s t-test, n values are individual cells from ≥ 2 biological repeats. (TIF) [file ppat.1009882.s008.tif]

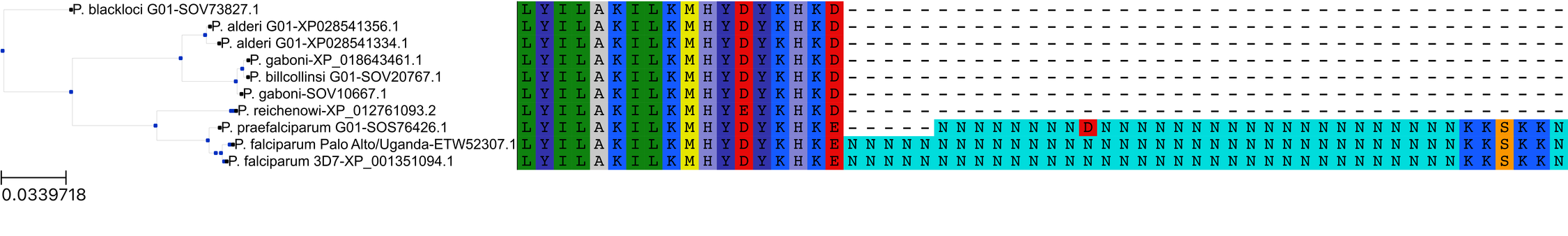

Supplement: S9 Fig — A phylogenetic tree and multiple sequence alignment calculated using the NCBI Standard Protein BLAST with a full-length PF3D7_0301700 (P. falciparum 3D7-XP_001351094.1) amino acid query. Sequences assembled in bioprojects accession: PRJNA329100, ID: 329100 and accession: PRJEB13584, ID: 445524. A hierarchical tree and color-coded sequence alignment were visualized using the ETE toolkit. NCBI provided organism names were replaced with taxa names and identical sequences were trimmed. Only the final 58 amino acids are displayed (the transmembrane domain to the end of the C-terminus queried). Distance scale indicated. (TIF) [file ppat.1009882.s009.tif]

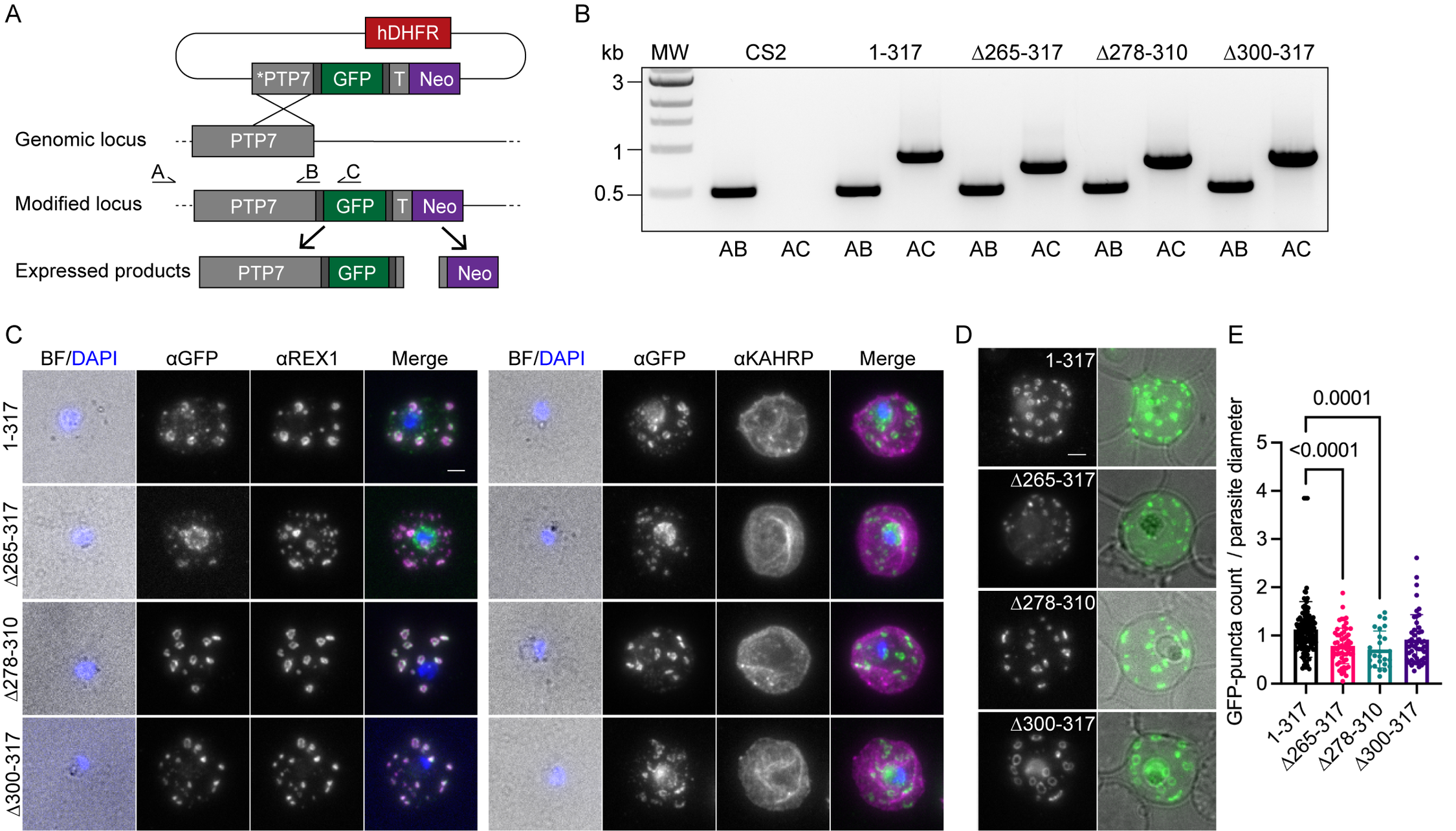

Supplement: S10 Fig — (A) Schematic illustrating selection linked integration of GFP at the 3’ end of the PTP7 locus. Dark gray rectangles: linkers; T: T2A skip peptide; Neo: neomycin selectable marker; hDHFR selectable marker; *PTP7: PTP7 homology region with 5’ stop codon; crossed lines: homologous cross over event; letters (A-E) and half arrows: Primer locations. (B) Confirmation of correct integration of the PTP7-GFPsand plasmid into the endogenous locus to generate cell lines introduced in Fig 5. (C) Indirect immunofluorescence assays of paraformaldehyde fixed infected RBCs probed with the antibodies displayed. Scale bar, 2 μm. (D) Live cell microscopy, native GFP fluorescence (green) merged with the BF (gray) image. (D-E) GFP-puncta quantification of live cell microscopy showing the mean GFP-puncta counts per cell normalized to parasite width (to control for age) for each of the truncations. Data displayed are mean ± SD (n = 110, 58, 26, 47 per cell line in the order displayed). P-values determined by Brown-Forsythe and Welch ANOVA tests and Dunnett’s multiple comparison test, n values are individual cells from ≥2 biological repeats. (TIF) [file ppat.1009882.s010.tif]

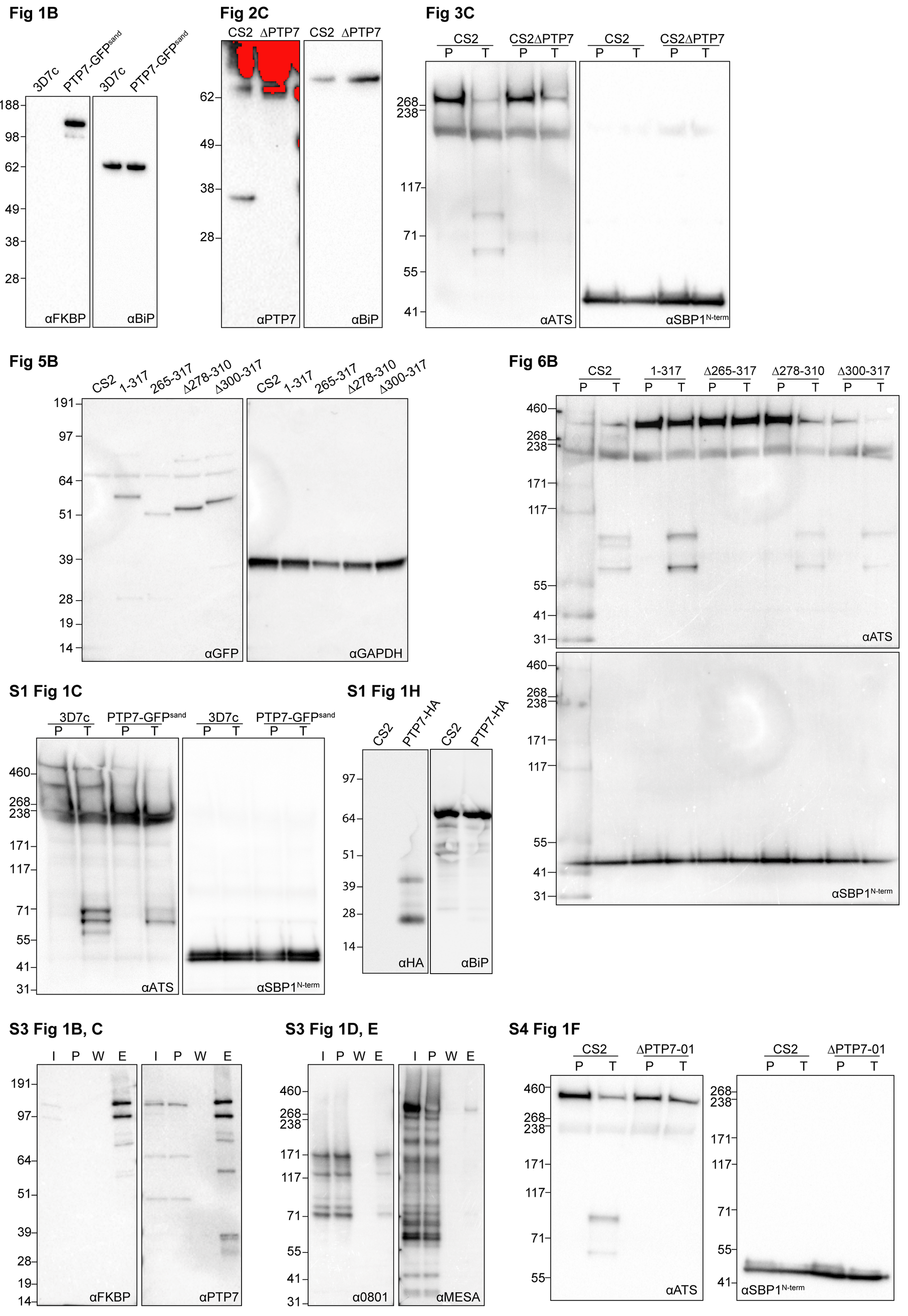

Supplement: S11 Fig — Saturated pixels indicated in red. (TIF) [file ppat.1009882.s011.tif]
