# Supplementary material for: Deletion of the Plasmodium falciparum exported protein PTP7 leads to Maurer’s clefts vesiculation, host cell remodeling defects, and loss of surface presentation of EMP1
Source: PLoS Pathog. 2022 Aug 5;18(8):e1009882. doi: 10.1371/journal.ppat.1009882 (PMC9385048; doi:10.1371/journal.ppat.1009882)
Supplement: S1 Table — (DOCX) [file ppat.1009882.s016.docx]

**S1 Table. Oligonucleotide appendix.**

| **Primer name** | **RE site** | **Sequence** |
| --- | --- | --- |
| PTP7-HR1_fw | AvrII | caggcgccag CCTAGG ggcaaaagatagtcaaaagaac |
| PTP7-HR1_rv | NcoI | atcgataact CCATGG aagaatataggaatacacaaagc |
| PTP7-HR2_fw | SpeI | agatcttcgg ACTAGT gattcacctgttttaaatgcattac |
| PTP7-HR2_rv | SacII | caatggccccttt CCGCGG cgataaccttcattcaaagttc |
| PTP7-sgRNA_top |  | TAAGTATATAATATTggttccaacacagtcacacgGTTTTAGAGCTAGAA |
| PTP7-sgRNA_bottom |  | TTCTAGCTCTAAAACcgtgtgactgtgttggaaccAATATTATATACTTA |
| PTP7-KO_ScrF |  | caatggaaaaagttttaagcctatt |
| PTP7-KO_ScrR |  | ggattaaacaaatatcataagggatac |
| yDHODH_ScrF |  | atgttcaaatgcatccttac |
| yDHODH_ScrR |  | ttagcttatgtcaactctatcaatag |
| SLI-sand-PTP7_fw | NotI | GCGGCCGCtaagaattaataaatccttagc |
| SLI-sand-PTP7_rv | AvrII | CCTAGGatttttttttgattttttattattattgttgtta |
| SLI-PTP7_ScrF |  | gacggaagaagaaataaagg |
| SLI-PTP7-Ntrm_rv | NheI | aatt gctagc tgcatttaaaacaggtgaa |
| SLI-linker_ScrR |  | cagatcttgatctcaatcctg |
| SLI-GFP_ScrR |  | aatagtctcgacctgaacacc |
| SLI-PTP7_fw | NotI | actatagaatactc GCGGCCGC taagaattaataaatccttagc |
| SLI-TGD-PTP7_rv | MluI | agcagcacctctagc ACGCGT atttttttttgattttttattattattg |
| SLI-TGD-PTP7∆300-317_rv | MluI | agcagcacctctagc ACGCGT attattattgttgttgttattattattg |
| SLI-TGD-PTP7∆278-310_rv | MluI | agcagcacctctagc ACGCGT atttttttttgatttttt ctctttatgtttgtagtcat |
| SLI-TGD-PTP7∆265-317 rv | MluI | agcagcacctctagc ACGCGT tgctaatatataaagtgctat |
| HA_rv |  | tagtccgggacgtcgtacgg |
