# Supplementary material for: Deletion of the Plasmodium falciparum exported protein PTP7 leads to Maurer’s clefts vesiculation, host cell remodeling defects, and loss of surface presentation of EMP1
Source: PLoS Pathog. 2022 Aug 5;18(8):e1009882. doi: 10.1371/journal.ppat.1009882 (PMC9385048; doi:10.1371/journal.ppat.1009882)
Supplement: Attachment — Submitted filename: Response to reviewers.docx [file ppat.1009882.s017.docx]

***Response to reviewers***

***Part I – Summary***

We thank the reviewers for their summaries of the work presented in the manuscript and their constructive suggestions. We acknowledge that the model proposed was speculative; and not sufficiently well supported by the data. We have removed the model and all reference to it. Instead, we have expanded the discussion of alternative mechanisms to explain the observed phenotypes (page 13, lines 301-314). All other concerns have been addressed in detail below.

***Part II – Major Issues: Key Experiments Required for Acceptance***

*Reviewer #1: The authors try to propose a model in which PTP7, via the charged residues at the C-terminus that appear specific to some Plasmodium species, acts as a driving force for vesicle fission for trafficking of PfEMP1 to the RBC surface. While the charged residues at the c-terminus could indeed play a role in membrane biology, there is no data supporting this possibility and other explanations are not well discussed.*

We apologize for the lack of clarity around the discussion of our truncation series and our model. We hypothesized that the steric effects due to the unstructured asparagine-rich C-terminal region of PTP7 provide the driving force for protein dispersion and ultimately membrane bending and fission (page 13, lines 301-306). We did not posit that the charged residues at the C-terminus are a major contributor to the driving force for vesicle fission. We have rephrased the final section of the results to clarify that the whole C-terminal domain, not exclusively the charged residues at the C-terminal end, may be required for the protein’s function in virulence factor trafficking and host cell remodeling.

*No membrane association studies have been performed and neither has the protein, or parts thereof, been expressed recombinantly and tested in liposome assays for membrane association. Therefore, in my mind all the data is showing is that deletion of PTP7, and of the C-terminal domain, leads to vesicle accumulation. I think this is a very interesting phenotype, but we have not learned how PTP7 plays a role in this. Because PTP7 also interacts with other proteins as shown by Co-IP, vesicle accumulation could also be an indirect effect and the positive amino acids are important for protein-protein interactions.* *These alternative explanations are not sufficiently discussed in the paper and the statement that PTP7 is a driving force in vesicle fission is not supported.*

Reviewer 1 is correct. In the absence of further studies testing the role of proteins identified by co-IP or *in vitro* studies testing the effect/association of recombinantly expressed regions PTP7 on liposomes, we do not know how PTP7 plays a role in vesicle accumulation at the Maurer’s cleft. We have expanded the limitations of our study in the discussion and address the possibility that PTP7 acts indirectly in virulence factor trafficking and host cell remodeling (page 14, line 307-314).

*Finally, the authors say that the additional amino acids are only present in some Plasmodium species and say this is maybe related to increased virulence. I have some issues with this statement: 1) some of the laverania species genome assemblies may not be great in the regions where PTP7 lies. It would be surprising if only Pg (the most distantly related Plasmodium species to Pf) carries the extension, and none of the other species. This should be carefully examined as the authors speculate that this extension is responsible in parts for increased virulence of Pf. It also is quite surprising that PTP7 is conserved across the Plasmodium species and the non-conserved extension plays a role in a fundamental process of vesicle fusion/ or fission. What is the role of the rest of the protein then in the other species? One could test this by deleting PTP7 in Plasmodium knowlesi, but I understand this may not be easily possible.*

Reviewer 1 has raised a very important point. We have scrutinized the literature and the quality of available datasets. The non-*P. falciparum* sequences are from two whole genome sequencing bioprojects (accession: PRJNA329100, ID: 329100 and accession: PRJEB13584, ID: 445524) wherein the sequence coverages range from 90x to 350x which are robust.

Additionally, we confirmed the reviewer’s suggestion that the only species carrying the extended C-terminus is *P. praefalciparum* which is the most closely related species to *P. falciparum*. The original multiple sequence alignment in S9 Fig used organism names as provided by NCBI. These taxa names have since been updated to their formal genus species names. Considering that *P. praefalciparum* is closely related, it is not surprising that the two species have more similar sequences than other Laverania species. We have updated the text (page 13, line 290-295) and the S9 Fig legend (page 34, lines 956-963).

Reviewer 1 suggests that PTP7 deletion studies in *Plasmodium knowlesi* might be insightful to the role of PTP7 homologues that lack the C-terminal extension. We appreciate Reviewer 1’s recognition that the suggested *P. knowlesi* studies would be very challenging; in particular, because there is no identified locus in *P. knowlesi* with synteny to PTP7.

*In conclusion I think the authors provide a solid piece of experimental data but discuss the potential function of PTP7 well beyond experimental evidence. I think they should delete the model they show as it lacks sufficient data to support it and include in the discussion alternative explanations for the PTP7 phenotype (indirect effects). Alternatively, the authors could decide to invest some more time in showing membrane association of PTP7 via the polybasic stretch to support their key conclusion about the mechanism by which PTP7 functions.*

Reviewer 1 has offered two options, to remove the speculative model in Fig 6F or to invest time in experiments dissecting the membrane association between the PTP7 C-terminal polybasic motif and liposome membranes. We have opted to remove the model and have provided additional text clarifying our original hypothesis that the low complexity C-terminal region generates steric hindrance that could lead to a biophysical preference for curved membranes (page 13, lines 303-306).

*For sure the speculation on the evolution of PTP7 in some but not all Plasmodium species, and claims about virulence need to be supported by quality checks on the genome data.*

See answer above.

*Reviewer #2: Two key issues need to be addressed to support the authors’ conclusions.
PTP7 localisation experiments are conducted solely by analysing PTP7 fusion proteins, particularly the FKB-GFP fusion. This protein is 4 to 5 times larger than the wild type PTP7 protein, due to the presence of the reporter moieties. An independent assessment of the sites and dynamics of PTP7 localisation is needed to confirm conclusions obtained with the fusion proteins and to clear concerns that trafficking and/or accumulation to specific compartment is affected by non PTP7 sequences.*

*PTP7 specific antibodies have been produced for this work, used only in western blot analysis; they (or additional ones in case they don’t work in parasite immunostaining) should be used to investigate the subcellular trafficking and trafficking of the endogenous PTP7.*

PTP7 specific antibodies were produced and used for immunoblot analysis. As shown in the full-length blots, S11 Fig, and in our response, these antibodies recognize a band of ~37 kDa corresponding to PTP7 which is absent from our CS2∆PTP7 parasite line. Unfortunately, the antiserum cross-reacts with higher molecular weight species that are present in both the parent CS2 and CS2∆PTP7 cell lysates.

Immunofluorescence assays were performed on wildtype CS2 and CS2∆PTP7 parasites using this anti-PTP7 antisera. While the antisera recognized Maurer’s clefts, additional regions of labelling were observed in both the wildtype and CS2∆PTP7 parasites, which is likely due to the cross-reaction with other proteins which were identified as higher molecular weight species by immunoblotting.

Reviewer 2 makes an important point regarding the size of the FKBP-GFP tag used in the PTP7-GFP^sand^ construct. Although not explicitly mentioned in the previous version of the manuscript, the endogenously expressed PTP7-GFP construct described later in the manuscript exhibits a similar GFP distribution by live cell and indirect immunofluorescence microscopy (anti-GFP, S10 Fig) to the PTP7-GFP^sand^ fusion protein.

To address any concerns that the GFP/GFP^sand^ tag may have an influence on knob formation, Maurer’s cleft architecture, or EMP1 trafficking, we have performed experiments that are included in the manuscript and detailed below.

We include electron microscopy images which indicate no obvious change in knob density or cleft morphology in the PTP7-GFP parasite infected RBCs (Fig 6C far left image and Fig 5C far left image, respectively). CS2 control images are shown in (Fig 2F top panel and Fig 4 top panels, respectively).

Transmission electron micrographs comparing the parent and PTP7-GFP^sand^ to S1 Fig demonstrate that, qualitatively, the cleft ultrastructure is not disturbed (described in the main text, page 4, lines 88-90). We performed a trypsinization assay to confirm that the presentation of EMP1 at the RBC surface is not compromised in the PTP7-GFP^sand^ infected RBCs (described on page 4, lines 86-88).

Further, indirect fluorescence microscopy images of other exported markers which show similar localization patterns between the wildtype and PTP7-GFP^sand^ parasite infected RBCs (Fig 1D, G, and S2 Fig, (described on page 5, lines 94-100).

For these reasons, we believe it is valid to rely on the GFP^sand^ and GFP tagged cell lines for our analyses. In an effort to address the reviewer’s points, the potential issue associated with using the large FKBP2x-GFP tag has now been mentioned, along with our controls validating the use of this cell line.

A figure containing an immunoblot (CS2 vs ∆PTP7) and indirect fluorescence assays using the PTP7 antibody and additional images of PTP7-GFP and GFP^sand^ infected RBCs has been provided to the editor and reviewers. We feel this data does not add to the manuscript but are happy to include this figure if the editor and reviewers request it.

*The gene ablation analysis needs to be confirmed either by generation of an independent KO line and/or by functional complementation of the existing disrupted line by expression of a wild type PTP7 protein.*

We have now included data for an independent CS2∆PTP7 cell line. We performed external scanning electron microscopy on infected RBC, revealing the same phenotype as exhibited in the first CS2∆PTP7 cell line. PCR validation of the independent CS2∆PTP7 cell line and external scanning electron micrographs have been added to S4 Fig and referenced in the results section (page 6, line 136-137).

*It is assumed that destibilization of the FKB-GFP fusion have been unsuccessful.*

The FKBP constructs used in this work are designed to perform knock sideways experiments and are not used as destabilization domains. Briefly, the FKBP domains will dimerize with an FRB domain in the presence of rapamycin. The FRB is expressed on a separate plasmid fused to a mCherry reporter and a mislocalising sequence. In our work we did not co-transfect our parasite lines with a mislocalising plasmid, and only used this plasmid for C-terminal GFP tagging. This system is described in detail in Birnbaum *et al*, 2017.

*Reviewer #3: (No Response)*

***Part III – Minor Issues***

*Reviewer #1: (No Response)*

*Reviewer #2: It is unclear whether the constructs producing the progressively deleted versions of the PTP7-GFP fusion are targeted to the ptp7 locus, ablating endogenous PTP7 expression, or elsewhere in the genome. Molecular confirmation of the chromosomal integration of these construct is needed.*

To address this point, we have updated the main text and methods to clearly state that all PTP7 constructs are integrated into the PTP7 locus (page 15, line 332; page 16, line 357). PCR validation has been added to S10 Fig (Page 34, lines 965-970).

*Reviewer #3: 1. Were the microscopy experiments blinded before quantification?*

Where applicable, object validation was performed manually in a blind manner. The methods have been updated to reflect this (page 20, lines 461-463).

*2. How many times was the GFP-trap pulldown and mass spectrometry performed?*

*3. The S3 Fig. mentions 3-fold enriched proteins. How was the 3-fold abundance determined?*

To address minor points 2 and 3 raised by Reviewer 3, we have edited the GFP-Trap pulldown methods and figure legend to make it clear that the pulldown and mass spectrometry was performed twice and that 3-fold enriched proteins were determined by fold enrichment of significant peptides relative to the parental line controls (page 18, lines 409-410; page 33, line 908-915).

**Characterization of PTP7 antisera and comparison of PTP7-GFP parasite lines.** (A) Immunoblot of CS2 and ∆PTP7 cell lysates probed with αPTP7 antibodies. (B) Indirect immunofluorescence microscopy of CS2 and ∆PTP7 infected RBCs stained will αPTP7 (green) and MAHRP1 (magenta) antibodies. Green arrows: nonspecific parasite staining; blue arrows: nonspecific staining of puncta in the infected red blood cell. (C-E) Indirect immunofluorescence microscopy of PTP7-GFP^sand^ and 1-317-GFP (full length PTP7-GFP, green), probed with αMAHRP1 and αREX1 (magenta) as shown. Bright field (BF) and DAPI stained DNA (blue) images are merged. Scale bar, 2 µm.
