# Supplementary material for: Deletion of the Plasmodium falciparum exported protein PTP7 leads to Maurer’s clefts vesiculation, host cell remodeling defects, and loss of surface presentation of EMP1
Source: PLoS Pathog. 2022 Aug 5;18(8):e1009882. doi: 10.1371/journal.ppat.1009882 (PMC9385048; doi:10.1371/journal.ppat.1009882)
Supplement: Attachment — Submitted filename: PTP7_resubmission02_rebuttal-letter_v03.docx [file ppat.1009882.s018.docx]

**Detailed response to reviewers**

*Editors and two previous reviewers have scrutinized your revised version of the manuscript. A reviewer remains unsatisfactory with the revisions in the Results Section, with still uncertainties about endogenous PTP7 localization and function. We all agree that data are not convincing enough in the revision to ascertain that PTP7 plays a role in vesicle accumulation at the Maurer’s cleft. Indeed, PTP7 interacts with many proteins and as stated by that authors, it may then act indirectly in virulence factor trafficking and host RBC remodeling. However, the topic is really interesting and we would like to give the opportunity to provide more experimental evidence and consider the assays proposed by the reviewer to clarify your data and justify a model at the end. In absence of additional results, we will not publish your work at this stage of the study.*

We thank the reviewers and editors for their detailed feedback and suggestions. We appreciate the reviews and the extra time granted to perform the requested experiments. As requested, we have now generated an endogenously tagged PTP7-HA cell line, which we have investigated in a side-by-side comparison with the PTP7-GFP^sand^ parasite line. We thank the reviewer for suggesting this. While the location of PTP7 is very similar in the two tagged lines, as outlined in more detail below there are some minor differences in the stage-dependent changes in location. Considering this, we have modified our conclusions. We are no longer confident that PTP7 moves through different compartments during maturation. Instead, we simply state that PTP7 co-locates with markers of several compartments within the protein export system.

In addition, we accept the reviewer’s comments regarding our previous conclusion that PTP7 is driving Maurer’s cleft vesiculation. Accordingly, we have now modified our conclusion, and added discussion points, highlighting multiple possible explanations for the observed vesiculation phenotype.

We have provided detailed responses to the reviewers’ questions below.

***Part I - Summary*** *Please use this section to discuss strengths/weaknesses of study, novelty/significance, general execution and scholarship.*

*Reviewer #1: I am happy with most of the implemented changes and congratulate the authors on a very nice study! But I still find 2 aspects of the discussion should be revisited (see below).*

We thank the reviewer for their kind comments and have addressed their comments below.

*Reviewer #2: The revised manuscript partially addressed some of the points raised in the review of the original submission.*

*In the discussion, the speculative mechanistic model of the PTP7 function proposed in the original submission has been removed. The sentence that “data provide intriguing evidence suggesting that the low complexity regions of PTP7 play a key role in providing the driving force for vesicle fission” is still an overstatement, but authors subsequently mention the possibility that PTP7 acts indirectly through an alternative mechanism, possibly involving protein-protein interactions.*

*Revision is less satisfactorily in the Results section.*

We accept the Reviewer’s point. We have now further modified the discussion and removed the statement that “the low complexity regions of PTP7 play a key role in providing the driving force for vesicle fission”. In addition, we have removed other statement that suggest that PTP7 *controls* vesiculation and instead explored several possible explanations, including indirect effects. We have also modified the title of the manuscript which now reads “Deletion of the *Plasmodium falciparum* exported protein PTP7 leads to Maurer’s clefts vesiculation, host cell remodeling defects and loss of surface presentation of EMP1”.

***Part II – Major Issues: Key Experiments Required for Acceptance***

*Reviewer #1: (No Response)*

*Reviewer #2: An independent determination of PTP7 localisation in the subcellular compartments of the parasitised RBC has not been provided to confirm the one obtained with the PTP7 fusion protein. Authors do explain that the anti-PTP7 antiserum used in Western blot cross reacts with several proteins, making it unsuitable to localise the wild type protein. If raising a new antibody with a tighter specificity is not feasible, a suggestion would be to produce a PTP7 fusion with a moiety structurally different from that used in the 3D7-PTP7-GFPsand parasites to confirm that both fusions show the same localisation pattern. In absence of the desirable independent confirmation, the evidence presented in the revised manuscript that Maurer’s cleft structure is unaffected in the 3D7-PTP7-GFPsand parasites is a weak argument to propose that the fusuion protein has the same localisation as the wild type PTP7.*

We thank the reviewer for their suggestion to generate an epitope tagged PTP7 cell line. We have generated transfectants expressing endogenous PTP7 tagged with 3xHA, in the CS2 parasite background (Fig 1 & S1 Fig). Airyscan super resolution microscopy was used to image immunofluorescently labelled samples to compare the HA and GFP^sand^ tagged PTP7 transfectants. In each case, tagged PTP7 is located at the periphery of REX1-labeled Maurer’s clefts (Fig 1F).

We also reanalysed the location of a Maurer’s cleft marker (REX1), an EDV marker (PTP2), and a J-dot marker (0801) in the two cell lines, at 4 time points across intraerythrocytic development. All data, including the original PTP7-GFP^sand^ data, was analysed and combined. As reported in the previous version of the manuscript, we confirmed that PTP7 exhibits partial co-occurrence with each of the compartment markers (S2 Fig).

Also as reported in the previous version of the manuscript, PTP7 exhibits a statistically significant increased co-occurrence with the J-dot marker, 0801, over the course of maturation.

However, we observed a minor difference between the two constructs upon reanalysis of the time courses for the co-occurrence of PTP7 with the Maurer’s cleft marker, REX1 and the EDV marker, PTP2 (S2 Fig). There is some variability between the samples; and we are no longer confident to interpret these minor changes as physiologically significant. We have modified the text accordingly (lines 123-129).

The text now reads: “To confirm the presence of PTP7 in different locations in the RBC cytoplasm, dual labelled immunofluorescence microscopy using antibodies recognizing GFP or HA and antigens located in different compartments were performed; and the co-occurrence at each location analysed at 4 timepoints across development (S2 Fig). Image analysis reveals ~40% co-occurrence with the Maurer’s cleft markers, MAHRP1 and REX1, as well as with and EMP1; but also reveals ~30% co-occurrence with the Maurer’s cleft/EDV-associated protein PTP2 (S2 Fig). Interestingly, the J-dot protein, 0801, shows an increasing level of co-occurrence with PTP7 as the parasite matures (S2 Fig).”

We thank the reviewer for suggesting these experiments that provide more confidence in our general conclusion that PTP7 is associated with different export compartments.

*The second argument, that PfEMP1 translocation and exposure on the RBC membrane is not affected in the 3D7-PTP7-GFPsand parasites is stated by the authors (line 88) but the supporting evidence (Fig. S1) shows qualitative and quantitative differences in the pattern of the surface exposed PfEMP1 diagnostic bands obtained in 3D7 vs 3D7-PTP7-GFPsand.*

We apologize for the confusion regarding our inclusion of the EMP1 trypsin cleavage data. Our intention with the inclusion of this data was to show that EMP1 is trafficked to the RBC membrane in the PTP7-GFP^sand^ parasites and that these parasites do not have an underlying trafficking defect. The differences in the appearance of the trypsin cleavage products in these blots is due to the cell line switching to the expression of a different PfEMP1 variant to that of the control cell line, a common occurrence for 3D7 parasites.

We have now modified the text to make this point clear (lines 88-90). The text reads: “A trypsin cleavage assay confirmed that EMP1 is still trafficked to the RBC surface in this line, albeit with fainter full length and trypsinized EMP1 fragments, possibly due to a switch in EMP1 variant expressed (S1 Fig).”

*The other point, i.e. to confirm the phenotype of the ptp7 gene deletion in an independent KO clone or by complementation of the one described, has been partially met. Production of an independent ptp7 KO clone is presented, but only two phenotypes are shown (Fig. S4) of those described to support the conclusions on PTP7 function, namely the reduced and dispersed presence of knobs on the surface of infected RBCs by scanning EM and the punctuate fluorescence staining obtained with the anti-KAHRP antibodies on the RBC surface.*

We have now provided data from two additional assays demonstrating the significant reduction of EMP1 from the surface of the independent knockout of *ptp7* (S4 Fig). The text has been modified to reference this new data. Line 160: “A complete loss of surface labeling was observed in ∆PTP7-infected RBCs (Fig 3B, S4 Fig)”. Line 161-164: “After trypsinization of intact infected RBCs, characteristic cleavage fragments are observed, confirming the presence of surface displayed EMP1 (Fig 3C, S4 Fig). In contrast, no cleavage products are observed in the ∆PTP7-infected RBCs (Fig 3C, S4 Fig).”

These data, along with the knob phenotypes provided previously, indicate that the phenotypes observed in the initial knockout are reproducible in an independently generated *ptp7* knockout.

***Part III – Minor Issues: Editorial and Data Presentation Modifications***

*Reviewer #1: 1) The discussion of the PTP7 function I find is still reads one-sided, whereby the authors appear to strongly favor a model in which the c-terminus of PTP7 is directly causing vesicle fission. Again, I don’t think there is sufficient evidence for it and the statement should be made with caution. I would feel more comfortable with a* *statement like that: ”We currently do not understand how the c-terminus of PTP7 facilitates vesiculation at the Maurer’s clefts. One hypothesis is that it is the c-terminus itself, through an increased hydrodynamic radius, causes vesicle budding. However, this needs to be formally tested and alternative explanations, such as a role of the C-terminus in facilitating protein-protein interactions required for vesicle budding are equally likely”.*

We thank Review 1 for their thoughtful suggestions. We have added the suggested text and expanded the discussion of other prospective roles of PTP7 (lines 312-318).

The text reads: “One possibility is that the low complexity asparagine repeats increase the hydrodynamic radius of the C-terminal domain of PTP7 [51,52]. This increased hydrodynamic radius, in turn, may drive a preferential location at highly curved membrane structures, like the edges of Maurer’s clefts and vesicles, possibly crowding these microenvironments and/or recruiting other proteins to induce vesicle fission. However, this needs to be formally tested and alternative explanations, such as a role of the C-terminus in facilitating protein-protein interactions that are required for vesicle budding are equally likely.”

In addition, we have modified the text throughout the manuscript and changed the title to be more careful not to over-interpret the vesiculation and to include consideration of alternative explanations.

*2) The authors speculate that the C-terminal extension tracks with increased virulence of Pf and P. praefalciparum compared to other Lavarania species. But are they more virulent? I don’t think we know this. This is maybe one of the most intriguing questions of the paper and I think the authors miss a great chance to discuss their findings in the light of the evolution of var genes across the Laverania (*[*https://www.nature.com/articles/s41564-018-0162-2*](https://www.nature.com/articles/s41564-018-0162-2)*). Var genes are present in other Lavarania species and it begs the question why don’t they need the extension. I think it is better to say “we cannot currently explain the evolution of the c-terminus in the light of what we know about the evolution of var genes in the Laverania, but it may suggest that…”.*

We thank the reviewer for raising the question of virulence in relation to other Lavarania species. We have removed the discussion of virulence and added the text suggested by the reviewer along with additional discussion of relevant findings from Otto *et al* 2018. (lines 295-307).

The text reads: “Conservation of PTP7 in the *Laverania* subgenus may be linked to the expansion of virulence protein trafficking and/or host cell remodeling machinery in these species. An analysis of the currently available *Plasmodium* genome assemblies showed that the N-terminal domain of PTP7 is well conserved in *P*. *praefalicparum*, *P*. *reichenowi*, *P*. *gaboni, P*. *billcollinsi*, *P*. *alderi,* and *P*. *blackloci* orthologues, but the C-terminal asparagine repeats are found only in *P. falciparum* and *P. praefalciparum* [54,55] (S9 Fig). We cannot currently explain the evolution of the C-terminus in the light of what we know about the evolution of var genes in the *Laverania*, but it may suggest that the expansion of the PTP7 sequence preceded the relatively recent speciation of *P. falciparum* and *P*. *praefalicparum* [56]. One hypothesis is that the expanded C-terminus of PTP7 interacts with the ATS domain of EMP1, which is conserved in *P. falciparum* and *P*. *praefalicparum*, a suggestion that is supported by our truncation data showing that this region is required for EMP1 trafficking; however, additional experiments would be required to test whether the interaction of PTP7 with ATS is enhanced in the expanded PTP7 sequence.”

*Reviewer #2: The sentences “**minimal effect in parasite biology” and “no changes in the ultrastructure of the infected RBCs” (lines 87 and 89) are generic and imprecise to describe the results they refer to. For instance, the latter sentence specifically refers to the ultrastructure of MCs, described to be undistinguishable in the 3D7-PTP7-GFPsand line and in the wild type parasites.*

We have removed the statement about “minimal effect on parasite biology” and specified that comments with respect to ultrastructure refers to the Maurer’s cleft (lines 90-92). The section now reads “We confirmed that EMP1 is still trafficked to the RBC surface in this line by performing a trypsin cleavage assay (S1 Fig). Likewise, electron micrographs of the parasite infected RBC confirmed that the addition of the GFP^sand^ tag had no effect on the Maurer’s cleft ultrastructure (S1 Fig).”

We have also made some additional minor changes to improve the grammar or readability. We have provided a tracked changes document that illustrates the changes made.

We believe that the changes improve the manuscript and thank the reviewers for their efforts.
